# Innate immune signatures in the nasopharynx after SARS-CoV-2 infection and links with the clinical outcome of COVID-19 in Omicron-dominant period

**DOI:** 10.1007/s00018-024-05401-1

**Published:** 2024-08-22

**Authors:** Hyunkyung Cha, Chan Mi Lee, Sujin Kim, Chang Kyung Kang, Pyoeng Gyun Choe, Yoon Kyung Jeon, Hyeon Jae Jo, Nam Joong Kim, Wan Beom Park, Hyun Jik Kim

**Affiliations:** 1 Department of Otorhinolaryngology-Head and Neck Surgery, Soonchunhyang University College of Medicine, Cheonan, Korea; 2 Department of Internal Medicine, Seoul National University College of Medicine, 101 Daehak-ro, Jongno-gu, Seoul, 03080 Korea; 3 Department of Otorhinolaryngology, Seoul National University College of Medicine, 103 Daehak-ro, Jongno-gu, Seoul, 03080 Korea; 4 Sensory Organ Research Institute, Seoul National University Medical Research Center, Seoul, Korea; 5 Department of Pathology, Seoul National University College of Medicine, Seoul, Korea

**Keywords:** SARS-CoV-2, COVID-19, Omicron variant, Nasopharynx, Interferon, Interferon-stimulated genes

## Abstract

While severe acute respiratory syndrome coronavirus-2 (SARS-CoV-2) is characterized by impaired induction of interferons (IFNs) and IFN-stimulated genes (ISGs), the IFNs and ISGs in upper airway is essential to restrict the spread of respiratory virus. Here, we identified the prominent IFN and ISG upregulation in the nasopharynx (NP) of mild and even severe coronavirus disease 2019 (COVID-19) patients (CoV2+) in Omicron era and to compare their clinical outcome depending on the level of IFNs and ISGs. Whereas the induction of *IFNB* was minimal, transcription of *IFNA*,* IFNG*, and *IFNL*s was significantly increased in the NP of CoV2 + patients. IFNs and ISGs may be more upregulated in the NP of CoV2 + patients at early phases of infection according to viral RNA levels and this is observed even in severe cases. IFN-related innate immune response might be characteristic in macrophages and monocytes at the NP and the CoV2 + patients with higher transcription of IFNs and ISGs in the NP showed a correlation with good prognosis of COVID-19. This study presents that IFNs and ISGs may be upregulated in the NP, even in severe CoV2 + patients depending on viral replication during Omicron-dominant period and the unique IFN-responsiveness in the NP links with COVID-19 clinical outcomes.

**Supplementary Information:**

The online version contains supplementary material available at 10.1007/s00018-024-05401-1.

## Introduction

In response to severe acute respiratory syndrome coronavirus-2 (SARS-CoV-2) infection, humoral and cellular immune responses promote the clearance of virus in concert with reducing severe damage to the lungs [1]. However, it has been proposed that SARS-CoV-2 can effectively blind the local or systemic immune responses and impairment of adequate immune responses might be associated with detrimental outcomes of coronavirus disease 2019 (COVID-19) [2, 3].

This immune escape can give rise to rapid virus replication in upper airway, which eventually results in hyperinflammation in the lung [4–6]. After the onset of SARS-CoV-2 infection, the defense mechanisms of the upper airway lead to limit viral replication in most individuals and to prevent further disease progression [7]. These mechanisms may include innate immune responses that are produced constitutively or induced immediately following infection. The innate immune system serves as the first line of defense at the respiratory mucosa by producing interferon (IFN), by which the antiviral innate immune response has been traditionally assumed to be exclusively mediated [8, 9]. In particular, IFNs mediate the induction of IFN-stimulated genes (ISGs) in the respiratory epithelium and provide front-line protection against respiratory viruses, suppressing the initial spread from the upper airway [10, 11]. Multiple datasets have identified putative SARS-CoV-2 targets within the nasal mucosa, including subsets of ciliated, secretory, and goblet cells, and type II pneumocytes within the lung parenchyma [12, 13]. Although not all COVID-19-diagnosed (CoV2+) patients progress to severe lung diseases, failed antiviral immunity in upper airway which is the direct viral target, underlie and precede severe COVID-19 [14]. Therefore, a better understanding of IFN-related immune responses in upper airway provides insights into the efficient defense mechanism to prevent the spread of SARS-CoV-2 and to restrict viral replication. Histologically, the nasopharynx (NP) has characteristics that are similar to those of lymphoid tissue and can be regarded as a structure in which immune responses can be activated after SARS-CoV-2 infection [15]. In addition, virus-host encounters take place at NP lymphoid tissues, and efficient or balanced antiviral properties are essential in the NP to protect the respiratory tract from viral infection [16, 17]. We investigated the innate immune responses mediated by IFNs and ISGs in the NP after SARS-CoV-2 infection and sought to determine the association with the therapeutic outcome of COVID-19.

Here, we identify prominent IFN and ISG upregulation in the NP of mild and even severe-CoV2 + patients and COVID-19 patients with higher viral RNA level in their NP showed more induction of IFNs and ISGs at early phase of Omicron-dominant infection. Our data also characterize the transcriptional landscapes of innate immune signatures in the NP with a strong impact on disease prognosis following SARS-CoV-2 infection.

## Materials and methods

### Sample collection and clinical data

NPs were accurately observed using an intranasal endoscope to obtain better quality lymphoid tissues and NP swabs were performed by an ENT (Ear, Nose, and Throat) specialist. NP lymphoid tissues were collected from laboratory-confirmed COVID-19 patients (SARS-CoV-2) depending on their admission date (or on the date of infection confirmation) and the date of discharge (or 7 to 14 days after the confirmed date) (*n* = 30) between May 2022 and January 2023 during the Omicron era at Seoul National University Hospital. Even patients with mild COVID-19 could be hospitalized if there were concerns about progression to pneumonia due to risk factors such as old age, or if subjective symptoms of COVID-19 were aggravated. The NP swabs from healthy controls were obtained during nasal surgery, which was performed before the COVID-19 pandemic (*n* = 10). These subjects had septoplasty to improve nasal obstruction under general anesthesia, and had no history of upper airway infection with high fever, cough, sputum and dyspnea for at least 3 months prior to surgery. The severity of COVID-19 patients was defined by the COVID-19 Treatment Guidelines Panel, which specifically defined severe patients as those with room air oxygen saturation below 94% and requiring supplemental oxygen therapy [18]. We also collected demographic data, including sex, age, underlying diseases, number of vaccine doses received before infection, previous history of COVID-19, admission, the length of hospital stay due to COVID-19 infection (excluding admission days for other comorbid problems), abnormal chest X-ray findings due to SARS-CoV-2 infection, and duration of supplemental oxygen therapy for those who needed it (Table 1). We also collected demographic data and calculated Sequential Organ Failure Assessment (SOFA) scores to support analysis of factors for poor outcomes [19]. The patients who were not hospitalized were considered to have a SOFA score of 0.

**Table 1:** The demographics of control group and COVID-19 patients

Factors	Control (*n* = 10)	CoV+ (*n* = 60)
Sex (M: F)	10 (100.0):0 (0.0)	36 (60.0):24 (40.0)
Age (years)	55.5 (42.7; 64.8)	67.0 (54.5; 81.5)
Number of vaccine doses	0.0 (0.0; 0.0)	3.0 (2.0; 3.0)
Previous history of COVID-19	0 (0.0)	4 (6.7)
Hospitalization		53 (88.3)
Length of hospital stay (days)*		7.5 (6.0; 10.0)
Days from symptom onset to first sampling		1.5 (1.0; 3.0)
Days from symptom onset to second sampling		9.0 (7.0; 11.3)
Initial chest X-ray abnormality		33 (63.5)
O_2_ supplementation tool**		
NP		17 (56.7)
HFNC		7 (23.3)
MV		5 (16.7)
ECMO		1 (3.3)
Duration of supplemental oxygen therapy (days)		0.5 (0.0; 8.5)
SOFA score		1.0 (0.0; 3.5)
Underlying comorbidity		
DM	3 (30.0)	19 (31.7)
HTN	4 (40.0)	23 (38.3)
CKD	0	7 (11.7)
LC	0	4 (6.7)
COPD	0	3 (5.0)
CTD	0	1 (1.7)
Hematologic malignancy	0	1 (1.7)
Solid cancer	0	11 (18.3)
Ct value at admission		21.5 (15.5; 29.2)

Data are presented as median (Q1; Q3) or number (%). ECMO: extracorporeal membrane oxygenation, HFNC: high-flow nasal cannula, MV: mechanical ventilation, NP: nasal prong, DM: diabetes mellitus, HTN: hypertension, CKD: chronic kidney disease, LC: liver cirrhosis, COPD: chronic obstructive pulmonary disease, CTD: connective tissue diseases, Ct: cycle threshold. *Calculations are based on hospitalized patients only, **Calculations are based on severe patients only (*n* = 30)

### RNA extraction and real-time PCR

Total RNA was extracted using TRI reagent (Molecular Research Center, Inc., Cincinnati, OH, USA) and cDNA was synthesized from 1 µg of RNA with random hexamer primers and Moloney murine leukemia virus reverse transcriptase (Enzynomics, Daejeon, Republic of Korea). Amplification was performed using the TaqMan Universal PCR Master Mix (Applied Biosystems, Foster City, CA, USA) according to the manufacturer’s protocol. Briefly, amplification reactions had a total volume of 12 µl and contained 2 µl of cDNA (reverse transcription mixture), oligonucleotide primers (final concentration of 800 nM), and TaqMan hybridization probe (200 nM). Real-time PCR probes were labeled at the 5’ end with carboxyfluorescein and at the 3’ end with the quencher carboxytetramethylrhodamine.

To quantify the gene expression, cDNA was generated from cellular RNA. Human primers of *IFNA*,* IFNB*,* IFNG*,* IFNL1*,* IFNL2/3*,* IFNL4*,* C-X-C motif chemokine ligand 10 (CXCL10)*,* Tumor necrosis factor alpha (TNFA)*,* Interleukin 1 beta (IL1B)*,* Myxovirus resistance 1 (MX1)*,* Interferon induced protein with tetratricopeptide repeats 1 (IFIT1)*,* IFIT2*,* IFIT3*,* Radical S-adenosyl methionine domain containing 2 (RSAD2)*,* Ubiquitin specific peptidase 18 (USP18)*,* ISG15*,* Interferon alpha induced protein 27 (IFI27)*,* and Interferon induced protein 44 like (IFI44L)* (Applied Biosystem, Foster City, CA, USA) were used (Supplementary Table 1). Real-time PCR was performed using a QuantStudio (TM) 3 Real-Time PCR System (Applied Biosystems, Foster City, CA, USA). The thermocycling parameters were as follows: 95 °C for 20 s, and then 40 cycles of 95 °C for 1 s and 60 °C for 20 s. All real-time PCR data were normalized to the level of glyceraldehyde phosphate dehydrogenase mRNA to correct for variations between samples.

### RNA sequencing data processing

After isolation of total RNA, RNA quality was assessed with a TapeStation4000 system (Agilent Technologies, Amstelveen, Netherlands). cDNA library construction was performed using the QuantSeq 3’ mRNA-Seq Library Prep Kit FWD (Lexogen, Inc., Austria) according to the manufacturer’s instructions. In brief, total RNA was prepared and an oligo-dT primer containing an Illumina-compatible sequence at its 5’ end was hybridized to the RNA; reverse transcription was then performed. After degradation of the RNA template, second strand synthesis was initiated by a random primer containing an Illumina compatible linker sequence at its 5’ end. The double-stranded library was purified using magnetic beads to remove all reaction components. The library was amplified to add the complete adapter sequences required for cluster generation. The finished library was purified from PCR components. High-throughput sequencing was performed as single-end 75 sequencing using a NextSeq 550 (Illumina, Inc., USA). The FASTQ files were subjected to adaptor trimming, low quality reads removal, and short reads removal using BBDuk [20]. All clean data were mapped to the human genome hg19 with default parameters.

### Differential expression and enrichment analysis

QuantSeq 3’ mRNA-Seq reads were aligned using Bowtie2 [21]. Bowtie2 indices were either generated from a genome assembly sequence or the representative transcript sequences for aligning to the genome and transcriptome. The alignment file was used for assembling transcripts, estimating their abundance, and detecting the differential expression of genes. Differentially expressed genes were determined based on counts from unique and multiple alignments using coverage in Bedtools [22] The Read Count (RC) data were processed based on the median of ratios and regularized log transformation normalization method using DESeq2 package version 1.40.1 within R using Bioconductor [23, 24]. Differentially expressed gene (DEG)s were determined with the threshold adjusted p value < 0.05 and absolute logged fold-change (Log2FC) ≥ 2 using the Bioconductor DESeq2 package [23]. In presenting the individual case of SARS-CoV-2, the fold change of each gene was calculated as the ratio of normalized gene expression of each SARS-CoV-2 individual to the mean expression of Healthy. The immune cell fraction was inferred using CIBERSORTx, which is a method for in silico flow cytometry. We used LM22 (consisting of 547 genes) as a reference signature matrix file for profiling 22 functionally defined human immune cell types [25]. Cell composition analysis was performed on raw read counts per gene converted into the number of transcripts per million. We also selected a batch correction option with 100 permutations. For the functional enrichment analysis of Upregulated DEGs, Biogrid Protein-protein interaction (PPI) networks and GO-BP (Gene Ontology-Biological Process) analysis were used to identify active subnetworks with R package pathfindR version 2.0.0. [26]. Subnetworks were identified via “Bonferroni” method and filtered by the enriched terms by adjusted p value < 0.05.

### Immunohistochemistry

1 × 1 cm sized-NP tissue was collected from 21-year-old male during endoscopic sinus surgery under general anesthesia and histologic examination of NP with hematoxylin and eosin staining was performed.

### Statistical analysis

Normality was determined using the Shapiro–Wilk test, Kolmogorov-Smirnov test, as well as considering sample number, skewness and kurtosis. The equality of variances was assessed with Levene’s test. Pearson × 2 or Fisher exact test was used to compare categorical variables between groups, and continuous variables were analyzed using the *t*-test or Mann-Whitney test. Two-sample *t*-testing was used to analyze the bulk-seq data. Correlation analysis was performed using point biserial correlation for categorical(binary) variables and Pearson correlation for continuous variables. Multiple linear regression with age was used for continuous dependent variables to examine factors that might influence outcome after controlling for demographic differences (age). Variables were normalized by applying log (1 + x) data transformation. p value < 0.05 indicated statistical significance. All statistical analysis was performed using R (version 4.1.2; R Foundation for Statistical Computing, Vienna, Austria).

## Results

### Histologic characterization of the lymphoid tissue of the human nasopharynx

**Fig. 1:**
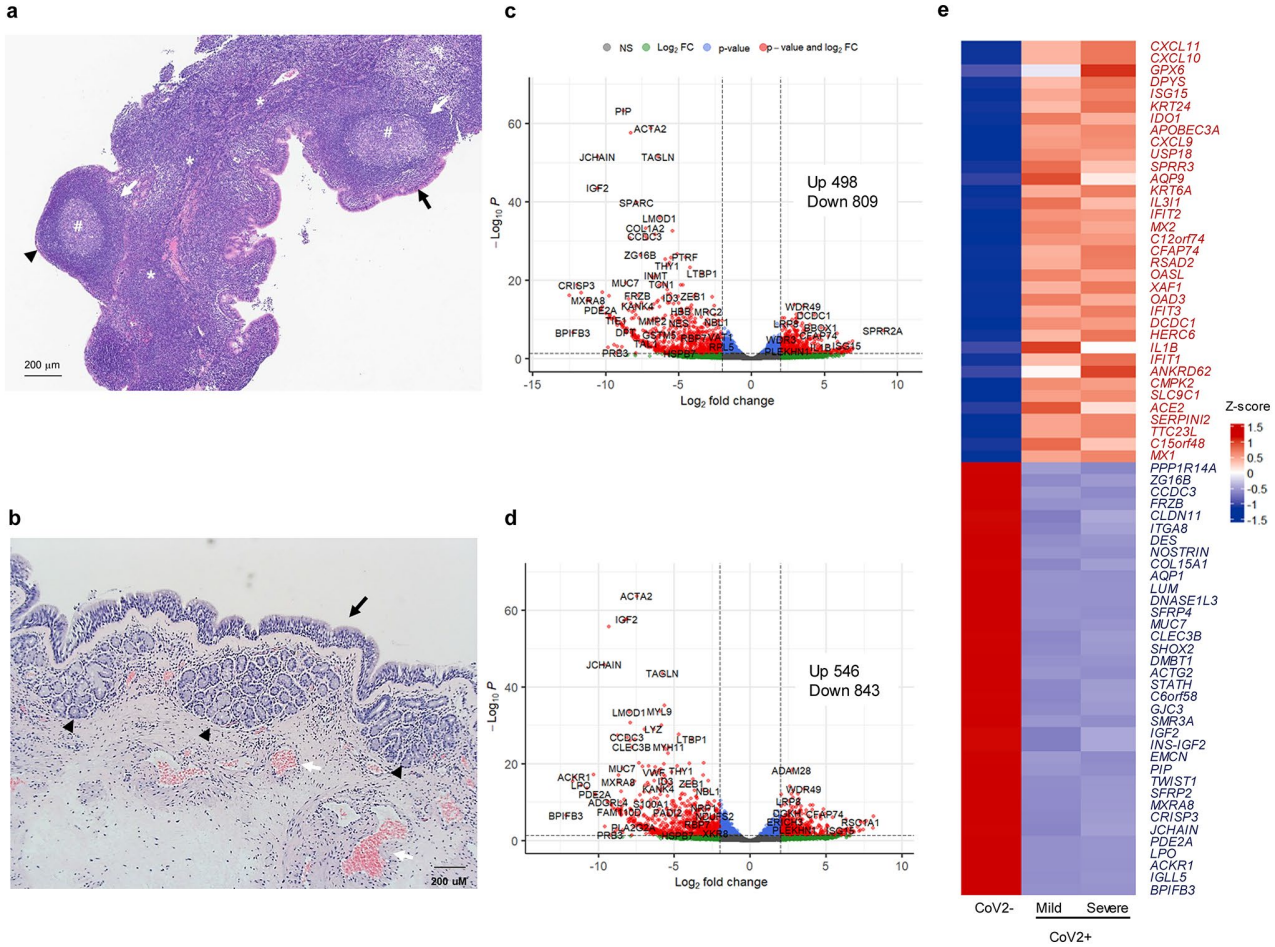
Histologic findings of NP and nasal mucosa and differentially expressed genes and cell proportions in RNA-seq datasets of CoV2 + patients. (**a**) The patient’s nasopharyngeal lymphoid tissue was observed and sampled through an intranasal endoscope by an ENT specialist. The histologic structure of hematoxylin and eosin–stained NP lymphoid tissue. Pseudostratified columnar epithelium (black arrow), squamous epithelium (black triangle), enlarged lymphoid follicles (white arrow), germinal center (#), and intercellular T cell zones (*) were characteristic of NP lymphoid tissue. Scale bar; 100 µM. (**b**) Histologic structure of hematoxylin and eosin–stained nasal mucosa. Pseudostratified columnar epithelium (black arrow), submucous glands (black triangle), and venous sinusoid (white arrow) were characteristic of nasal mucosa. Scale bar; 200 µM. The histologic results are representative of NP tissues and nasal mucosa from three adults. (**c**) Volcano plot of regulated genes on nasopharyngeal swabs of 3 mild-CoV2 + patients and 3 healthy controls. Cut-off values of differentially expressed genes are log2-fold change > |2.0| and adjusted p < 0.05. (**d**) Regulated genes on nasopharyngeal swabs of 3 severe-CoV2 + patients and 3 healthy controls. Cut-off values of differentially expressed genes are log2-fold change > |2.0| and adjusted p < 0.05. (**e**) Heatmap of average differentially expressed genes of controls, mild, and severe CoV2 + patients. The 35 most common upregulated and downregulated genes of CoV2 + patients compared with controls (*n* = 3 for each group)

First, a histologic result of NP biopsy from a 21-year-old male patient without history of SARS-CoV-2 infection was provided from the department of pathology in Seoul national university college of medicine (co-author YK Jeon) and investigated the histology of human NP lymphoid tissue. The histologic results revealed that a multilayered pseudostratified columnar epithelium and a squamous epithelium were observed at the surface of NP lymphoid tissue. This tissue showed distinctive histologic characteristics compared with nasal mucosa, and lymphoid follicles, germinal center, and intercellular T-cell zones were observed beneath NP epithelium (Fig. 1a). Histologic examination of nasal mucosa revealed a multilayered pseudostratified columnar epithelium, ciliated cells, and secretory cells. Both submucous glands and venous vessels were located beneath the nasal epithelium and were separated from the epithelium by a basement membrane (Fig. 1b). The germinal center, lymphoid follicles, and T-cell zone were not observed in the nasal mucosa. We estimated that the NP tissue had a unique structure similar to that of a lymph node and possessed a respiratory epithelium, suggesting that epithelial-derived innate immune responses could be activated when SARS-CoV-2 contacts the NP.

### DEGs and cellular composition in the NP of mild or severe COVID-19 patients

We identified the DEGs in the NP of CoV2 + patients through bulk RNA sequencing (bulk-seq) as well as the differences between mild (*n* = 3) and severe (*n =* 3) cases and compared the transcription profiles with NP of healthy subjects (those who tested negative for SARS-CoV-2 [CoV2−], *n =* 3). Bulk-seq data identified 554 upregulated and 869 downregulated DEGs in the NP of CoV2 + patients, with a cutoff value of (Log2FC) ≥ 2 and an adjusted p < 0.05. Among them, 498 upregulated and 809 downregulated DEGs were found in CoV2 + patients with mild disease compared to CoV2 − patients (Fig. 1c), and 546 upregulated and 843 downregulated DEGs were found in CoV2 + patients with severe disease (Fig. 1d). We found striking differences between CoV2 + and CoV2 − patients regarding the transcriptions of IFN-related innate immune responses in the NP and the top 35 upregulated DEGs in CoV2 + patients compared with the comparable DEGs in CoV2 − patients, with a focus on ISGs (Fig. 1e). The heterogeneity of individual sample data was shown in Supplementary Fig. 1. These findings indicate potent induction of ISGs in the NP following SARS-CoV-2 infection the induction of ISGs was also identified in the NP of severe-CoV2 + patients.

### Innate immune responses in the NP following SARS-CoV-2 infection

**Fig. 2:**
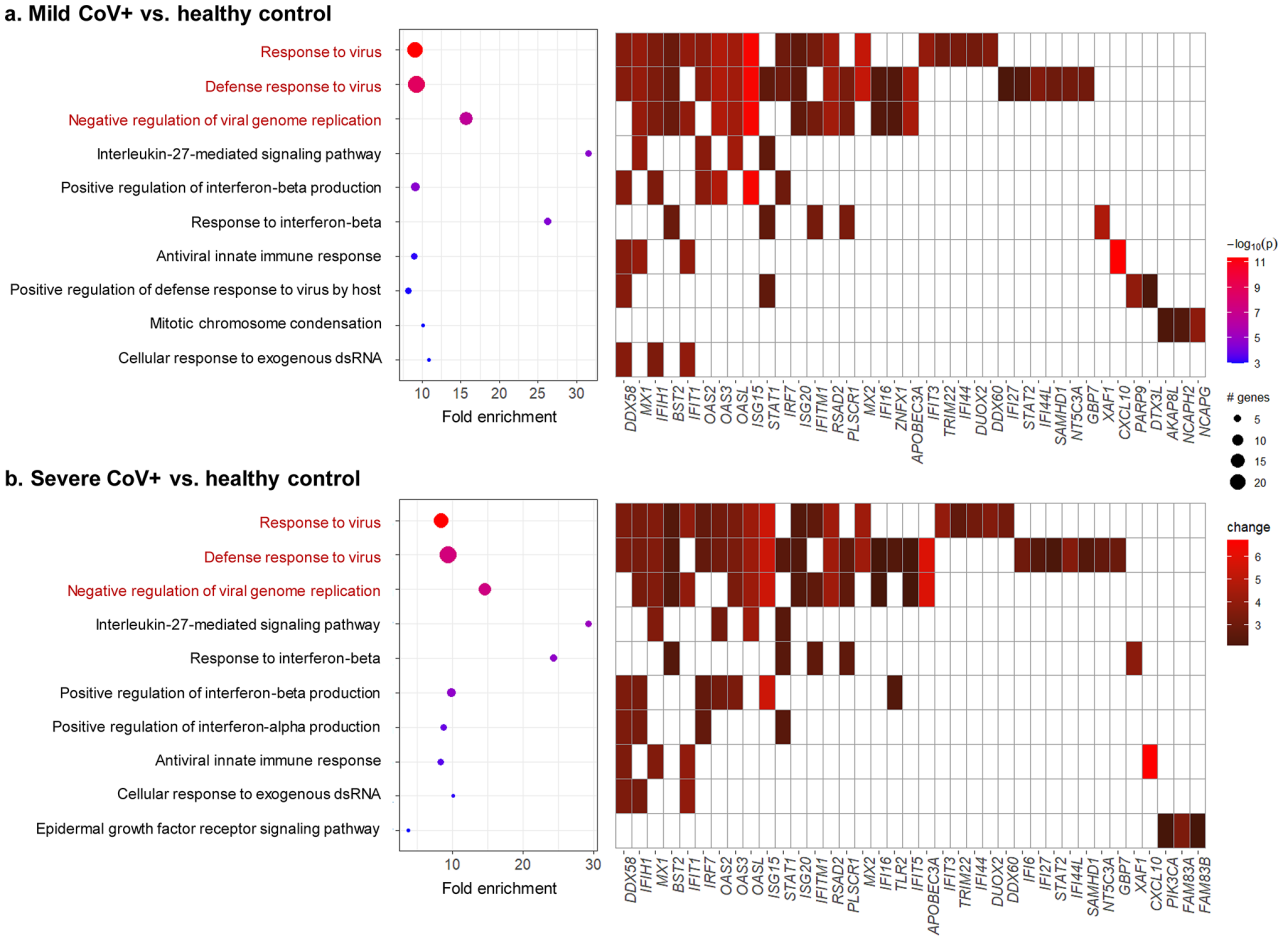
Enrichment analysis of CoV2 + patients compared to healthy controls using gene ontology–biological process gene sets (*n* = 3 for each group). (**a**) An enrichment chart and a term gene heatmap of mild-CoV2 + patients (*n* = 3). (**b**) An enrichment chart and a term gene heatmap of severe-CoV2 + patients (*n* = 3). Enriched terms were filtered by an adjusted p value < 0.05 (Bonferroni method)

As a next step, we performed active-subnetwork-oriented enrichment analysis using on GO-BP terms. Enrichment p values were adjusted with Bonferroni method and filtered the enriched terms by adjusted p value under 0.05 [26]. The GO categories of “response to virus” and “defense response to virus” were most significant in the NP of both mild- (Fig. 2a) and severe-CoV2 + patients (Fig. 2b) compared to controls. An analysis of the genes involved in the enriched terms of mild- (Fig. 2a) and severe-CoV2 + patients (Fig. 2b) showed common or distinct genes between terms. Transcription of diverse ISGs and IFN-related genes was elevated in the NP of mild and severe-CoV2 + patients, but no induction of IFNs was detected in bulk-seq data. In mild-CoV2 + patients, 23 DEGs were included in the “defense response to virus” GO category and 19 in the “response to virus” category compared with CoV2 − patients (Fig. 2b). In addition, 25 DEGs included in the “defense response to virus” category and 19 DEGs in the “response to virus” category was found in the NP of severe-CoV2 + patients (Fig. 2b). These included *IFI44L*, *MX1*, *MX2*, *RSAD2*, *IRF7*, *BST2*, and *APOBEC3A*, while *ISG15* showed the largest increase in the NP of both mild- and severe-CoV2 + patients.

**Fig. 3:**
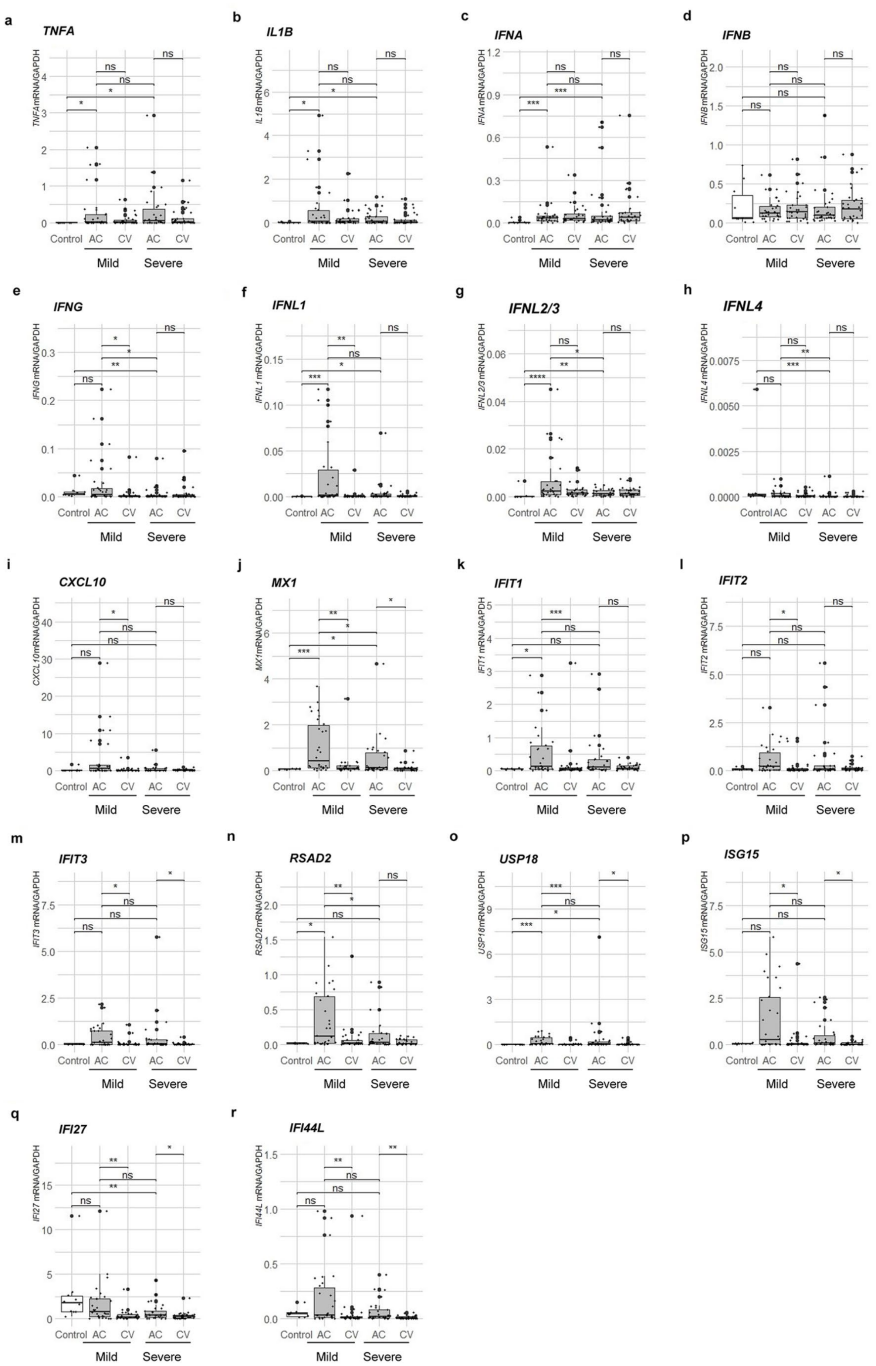
mRNA transcription levels of IFNs and ISGs, including (**a**) *TNFA*, (**b**) *IL1B*, (**d**) *IFNA*, (**d**) *IFNB*, (**e**) *IFNG*, (**f**) *IFNL1*, (**g**) *IFNL2/3*, (**h**) *IFNL4*, (**i**) *CXCL10*, (**j**) *MX1*, (**k**) *IFIT1*, (**l**) *IFIT2*, (**m**) *IFIT3*, (**n**) *RSAD2*, (**o**) *USP18*, (**p**) *ISG15*, (**q**) *IFI27*, and (**r**) *IFI44L*, were assessed in the control group, as well as in mild and severe patients during both the acute and convalescent phases (mean ± SEM; Mann-Whitney test; *n* = 10 for control, *n* = 30 for each CoV + group). All mRNA transcription levels were normalized to that of GAPDH. The significance levels are represented as follows: ^ns^ indicates p value > 0.05, * indicates p value < 0.05, ** indicates p value < 0.01, *** indicates p value < 0.001, and **** indicates p value < 0.0001

We then measured 18 cytokines, including IFNs and ISGs, in the NP of mild- (*n =* 30) or severe-CoV2 + patients (*n =* 30) at hospitalization (acute = AC) and discharge (convalescent = CV). PCR results showed that transcription of both *TNFA* and *IL1B* was elevated in the NP of mild-CoV2 + patients (p = 0.026 and 0.050) and severe-CoV2 + patients (p = 0.031 and 0.046) at AC, while mRNA levels were downregulated at CV (Fig. 3a, b). Expression levels of *IFNA*,* IFNL1*, and *IFNL2/3* were increased in the NP of mild-CoV2 + patients at AC and were lower at CV. In contrast, *IFNB* mRNA level was not induced in the NP of CoV2 + patients at AC and CV. Although the expression level was lower than in mild-CoV2 + patients, the mean mRNA levels of *IFNA*,* IFNL1*, and *IFNL2/3* were significantly higher in the NP of severe-CoV2 + patients at AC compared with that of the CoV2 − group, but *IFNL4* and *IFNG* expression levels were significantly decreased in severe patients compared with mild patients (p = 0.005, and 0.013, respectively) (Fig. 3c-h).

The mean mRNA levels of ISGs, including *MX1*, *IFIT1*, *RSAD2*, and *USP18*, were increased in the NP of mild-CoV2 + patients at AC (p < 0.001, 0.012, 0.017, and < 0.001, respectively), and no induction of ISGs was detected in mild-CoV2 + patients at CV (Fig. 3i-r). *MX1*, *RSAD2*, and *USP18* mRNA levels were relatively high in severe-CoV2 + patients at AC (p = 0.046, 0.400, and 0.012, respectively), but the transcription levels of *MX1* and *RSAD2* in severe patients were lower than those in mild-CoV2 + patients (p = 0.018 and 0.043). Transcription of *IFIT1* increased only in mild acute-phase CoV2 + compared with CoV2 − patients (p = 0.012). Although the mean mRNA level of *IFI27* was different from bulk-seq data, a decrease in transcription level was seen in the NP of severe-CoV2 + patients compared with CoV2 − patients, and no significant induction of *ISG15* and *IFI44L* was observed in the NP of mild- or severe-CoV2 + patients. Based on these findings, we estimated that significant induction of IFNs, except *IFNB* and ISGs including *MX1*, *IFIT1*, *RSAD2*, and *USP18*, occurred in the NP of mild-CoV2 + patients, and that IFN-related innate immune responses were activated in the NP of severe-CoV2 + patients at the onset of infection. However, it was difficult to find a correlation between the genes included in the downregulated GO categories and the immune response (Supplementary Fig. 2).

### IFN-related immune cells in the NP of CoV2 + patients

**Fig. 4:**
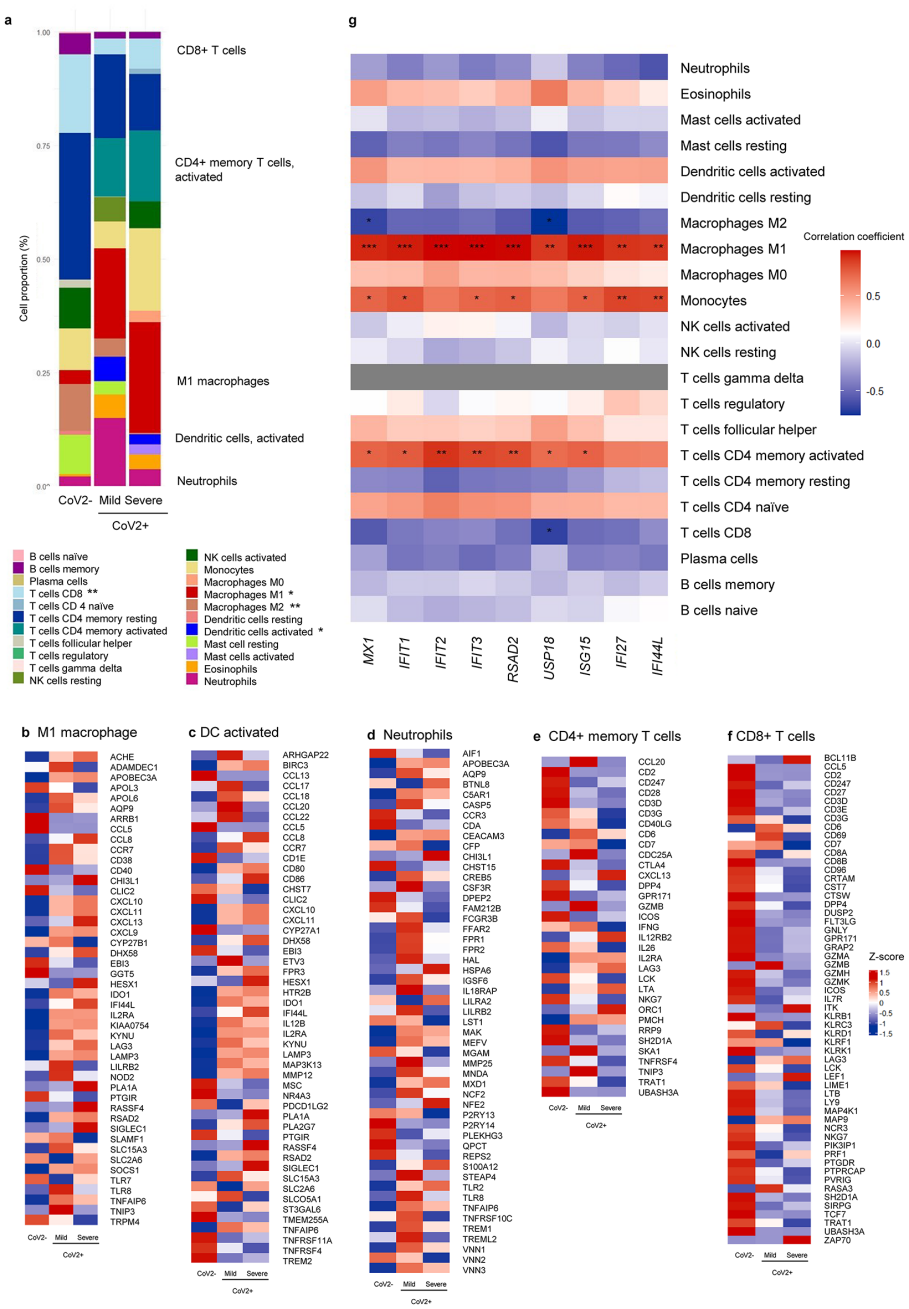
Average immune cell proportions and related DEGs among controls and CoV2 + patients (*n* = 3 for each group). (**a**) Average immune cell proportions for controls, mild-, and severe-CoV2 + patients (*t*-test). M1 and DCs are significantly elevated in CoV2 + patients. DEGs for (**b**) M1 macrophage, (**c**) Dendritic cells activated, (**d**) Neutrophils, (**e**) CD4 memory T cells (activated), (**f**) CD8 T cells according to LM22 were shown as heatmap. (**g**) Pearson correlation test between immune cell proportions of each sample and ISGs demonstrated the relevance of M1 macrophages and monocytes with ISGs. *MX1*,* IFIT1*,* IFIT2*,* IFIT3*,* RSAD2*,* USP18*,* ISG15*,* IFI27*, and *IFI44L* were log-transformed due to skewed distribution. The significance levels are represented as follows: * indicates p value < 0.05, ** indicates p value < 0.01, and *** indicates p value < 0.001

Last, we used the CIBERSORT algorithm to calculate immune cell proportions and compared gene expression associated with 22 types of immune cells in the NP of CoV2 + patients. We used t-test to compare fraction of each immune cell type between control and CoV + patients. A significant increase was evident in the transcriptional proportion of M1 macrophages (MФs) in the NP of mild and severe-CoV2 + patients compared with CoV2 − subjects (p* =* 0.026), and a significant decrease was evident in the transcription of M2 MФ (p = 0.005). In addition, expression of genes associated with CD4 + memory T cells, activated dendritic cells (DCs) was greater (p = 0.079 and 0.017) in the NP of mild and severe-CoV2 + patients, while that of neutrophils was higher among mild-CoV2 + patients (Fig. 4a). In contrast to these immune cells, the transcription of CD8 + T cells was significantly attenuated in the NP of mild- and severe-CoV2 + patients (p = 0.009). Heatmaps show the relative expression of genes related to M1 MФ, activated DCs, neutrophils, CD4 + memory T cells, and CD8 + T cells from the LM22 [27] in the NP of CoV2 + patients (Fig. 4b-f). When investigating the COVID-19 IFN response, we found that transcriptions of ISGs which were correlated with the prognosis of CoV2 + patients and were significantly associated with higher transcriptions of M1 MФ and monocytes -related genes in the NP of CoV2+ (Fig. 4g). These results suggested that the transcriptions of immune cells were altered in the NP of CoV2 + patients and IFN-related innate immune response might be characteristic in M1 MФ of the NP, when the is trying to control the viral infection in upper airway.

### Induction of IFNs and ISGs in the NP is correlated with the clinical outcome of COVID-19 treatment

**Fig. 5:**
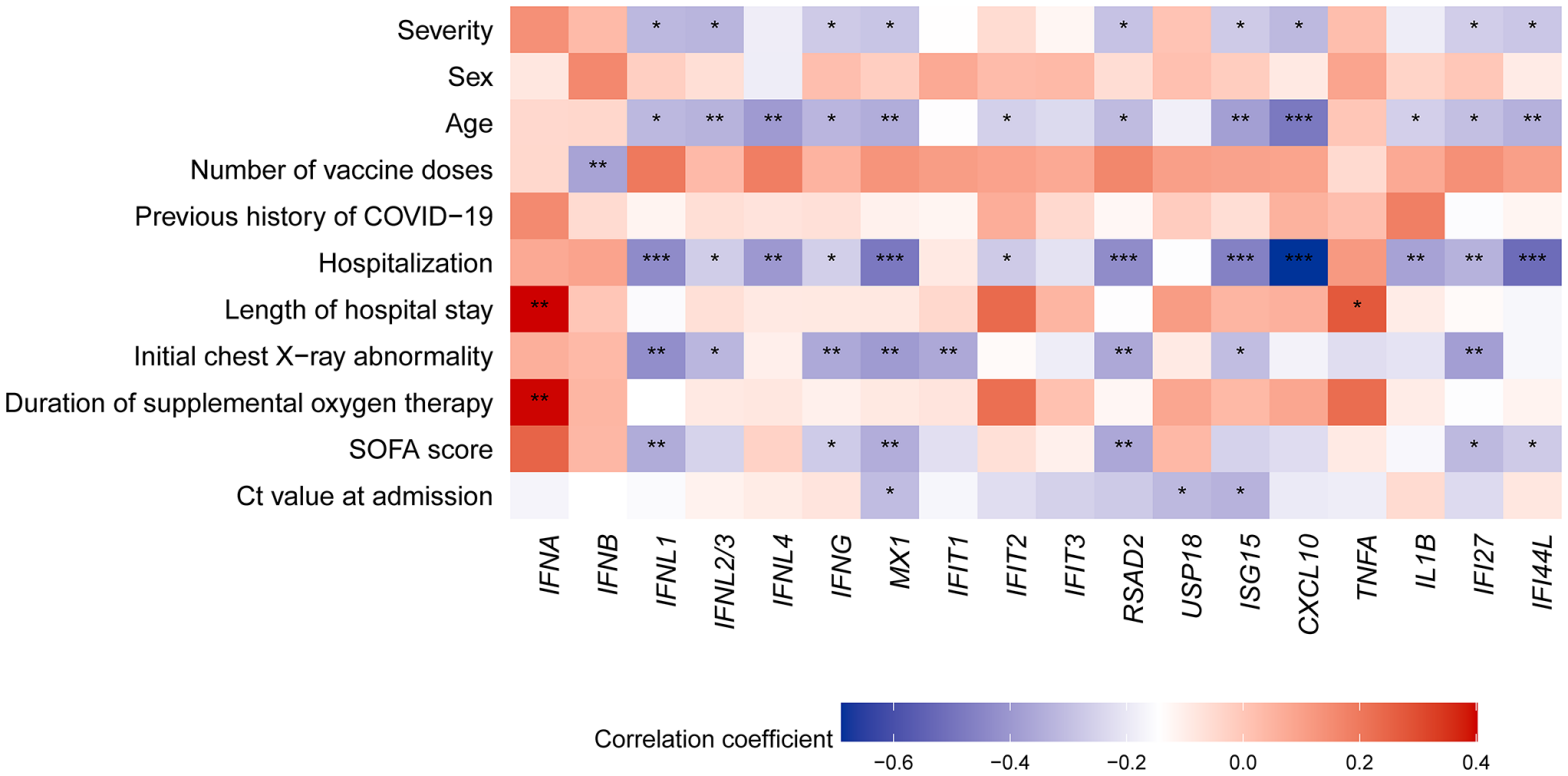
A heatmap of the correlations between clinical factors and markers of innate immune responses (*n* = 60). IFNs and ISGs were log-transformed for normalization. Pearson correlation test (point-biserial correlation test for binary variables) was performed. The significance levels are represented as follows: * indicates p value < 0.05, ** indicates p value < 0.01, and *** indicates p value < 0.001

To better understand whether the induction of IFNs and ISGs in response to SARS-CoV-2 infection of the NP is correlated with the prognosis of COVID-19, we explored the correlations between IFN and ISG transcription and clinical factors in CoV2 + patients. First, we identified dynamic parameters over the prognosis of CoV2+, including age, sex, number of vaccine doses, previous history of COVID-19, hospitalization, length of hospital stays, presence of initial chest X ray abnormalities, SOFA score, and the incidence of comorbid diseases (Table 2). Next, we analyzed the correlations between the clinical parameters of CoV2 + patients and IFNs or ISGs (Fig. 5, Supplementary Tables 2, 3). The results revealed that age in CoV2 + patients was significantly negatively correlated with mean mRNA levels of *IFNL1*,* IFNL2/3*,* IFNL4*,* IFNG*,* CXCL10*,* IL1B*,* MX1*,* IFIT2*,* RSAD2*,* ISG15*,* IFI27*, and *IFI44L*. Hospitalization had a significant negative correlation with *IFNL1*,* IFNL2/3*,* IFNL4*,* IFNG*,* MX1*,* IFIT2*,* RSAD2*,* ISG15*,* CXCL10*,* IL1B*,* IFI27*, and *IFI44L*. The initial chest X-ray abnormality in CoV2 + patients was negatively correlated with the expression of *IFNL1*,* IFNL2/3*,* IFNG*,* MX1*,* IFIT1*,* RSAD2*, ISG15, and *IFI27* (Fig. 5, Supplementary Tables 2, 3). The median duration of supplemental oxygen therapy of severe-CoV2 + patients was 8.5 days (Q1, 4.0 days; Q3, 15.0 days; mean ± standard deviation, 14.2 ± 23.2) (Fig. 5, Supplementary Tables 2, 3). SOFA score was negatively correlated with the expression of *IFNL1*,* IFNG*,* MX1*,* RSAD2*,* IFI27*,* IFI44L.* After adjusting for age (multiple linear regression), demographic value that showed a significant difference between mild and severe group, MX1 (coefficient: -0.455, p = 0.033) and RSAD2 (coefficient: -0.370, p = 0.024) expression levels significantly predicted the SOFA score (Table 3). We found that COVID-19 patients with significant induction of IFNs and ISGs in their NP showed relatively good clinical factors after treatment in the early stages of infection.

**Table 2:** The demographics of mild and severe COVID-19 patients

Factors	Mild (*n* = 30)	Severe (*n* = 30)	p
Sex (M: F)	18 (60):12 (40)	18 (60):12(40)	> 0.999
Age	63.5 (30.0; 70.0)	79.5 (64.0; 85.0)	< 0.001
Number of vaccine doses	3.0 (3.0; 3.0)	3.0 (2.0; 3.0)	0.860
Previous history of COVID-19	3 (10.0)	1 (3.3)	0.612
Hospitalization	23 (76.7)	30 (100.0)	0.011
Length of hospital stay (days)	7.0 (3.0; 8.0)	9.0 (7.0;12.0)	< 0.001
Initial chest X-ray abnormality	6 (27.3)	27 (90.0)	< 0.001
Days from symptom onset to first sampling	1.0 (1.0; 1.0)	3.0 (3.0; 5.0)	< 0.001
Days from symptom onset to second sampling	9.0 (8.0; 12.0)	8.0 (7.0; 11.0)	0.788
SOFA score	0.0 (0.0; 1.0)	3.0 (1.0; 5.0)	< 0.001
Underlying comorbidity			
DM	6 (20.0)	13 (43.3)	0.096
HTN	7 (23.3)	16 (53.3)	0.034
CKD	2 (6.7)	5 (16.6)	0.228
LC	2 (6.7)	2 (6.7)	> 0.999
COPD	0 (0.0)	2 (6.7)	0.237
CTD	0 (0.0)	1 (3.3)	> 0.999
Hematologic malignancy	0 (0.0)	1 (3.3)	> 0.999
Solid cancer	5 (16.7)	6 (20.0)	> 0.999
Ct value at admission	22.4 (15.4; 29.6)	21.1 (17.4; 26.5)	0.707

Data are presented as median (Q1; Q3) or (number (%)). DM: diabetes mellitus, HTN: hypertension, CKD: chronic kidney disease, LC: liver cirrhosis, COPD: chronic obstructive pulmonary disease, CTD: connective tissue disease, Ct: cycle threshold

**Table 3:** IFNs and ISGs related with SOFA score. Multiple linear regression adjusted for age and sex was performed. Log transformation was performed on IFNs or ISGs. IFNs or ISGs resulting in significant models are listed

Related IFNs and ISGs	Coefficients	p
*MX1*	-0.455	0.033
*RSAD2*	-0.370	0.024
*IFIT1*	-0.346	0.076
*ISG15*	-0.192	0.141
*IFI44L*	-0.226	0.167

IFN: interferon, ISG: IFN-stimulated genes, *MX1*: Myxovirus resistance 1, *RSAD2*: Radical S-Adenosyl Methionine Domain Containing 2, *IFIT1*: Interferon Induced Protein with Tetratricopeptide Repeats 1,* IFI44L*: Interferon Induced Protein 44 Like

### Innate immune signatures in the NP are associated with viral RNA level and the prognosis of COVID-19 treatment

**Fig. 6:**
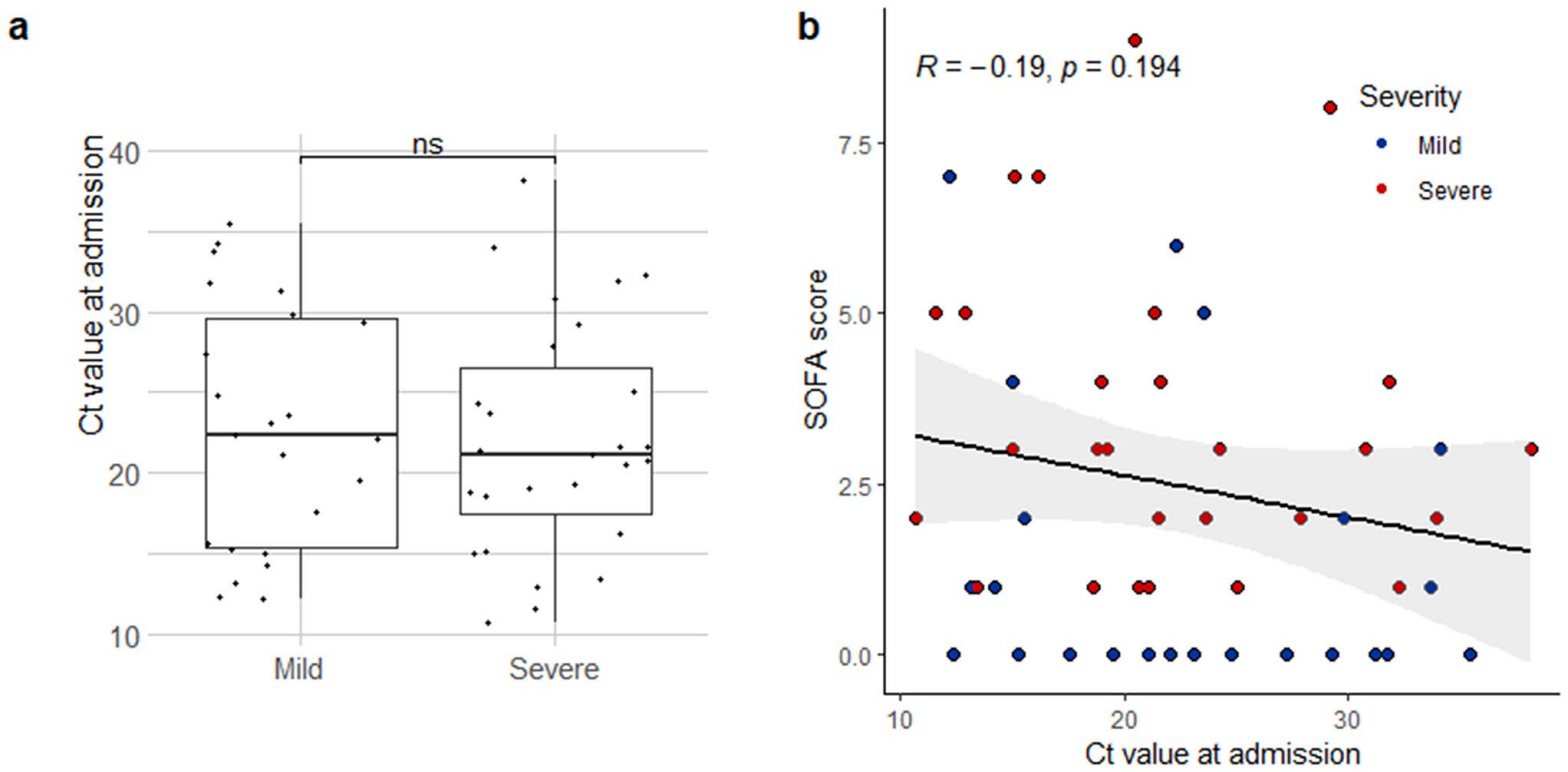
Relationships between the Ct value at admission and the severity and innate immune response markers (mild *n* = 23, severe *n* = 28). (**a**) The Ct value at admission showed no difference between mild and severe-CoV2 + patients. The 2-tailed *t*-test was used to compare each group. ^ns^ indicates p > 0.05. Error bars indicate the SEM. (**b**) There was no significant correlation between the Ct value at admission and SOFA score (Pearson test)

We investigated the correlation between Ct value at admission or mortality predictions using SOFA scores, which is one of the criteria for determining the severity of disease more definitely. The results of the SARS-CoV2 PCR performed on NP samples when COVID-19 was confirmed showed that the viral RNA level did not exhibit a direct association with the severity of COVID-19 (*t*-test, p = 0.707) (Fig. 6a) or with the SOFA score of COVID-19 patients (Pearson correlation, p = 0.194) (Fig. 6b). After adjusting for age (multiple linear regression), demographic value that showed a significant difference between mild and severe group, the Ct value at admission influences approximately 1.8% of the SOFA score (multiple linear regression, coefficient: -0.054, p = 0.263, adjusted R-squared: 0.018). However, Innate immune signatures such as *MX1*, *USP18*, and *ISG15* in the NP were associated with Ct value at admission of COVID-19 patients (Fig. 5, Supplementary Tables 2,3). The additional results revealed that the gene expression of *IFNL1*,* IFNG*,* MX1*,* IFIT2*,* IFIT3*,* ISG15*,* RSAD2*,* IFI27*,* IFI44L*, and *USP18* in the NP was negatively correlated with Ct value at admission (Table 4), and CoV2 + patients with relatively low expression of innate immune factors in the NP had more severe disease. Based on these findings, we determined that *MX1* and *RSAD2* levels were significantly higher in the NP of CoV2 + patients with higher viral RNA level at initial stage of infection and the induction of both IFNs and ISGs in the NP of CoV2 + patients may be closely correlated to the good prognosis of COVID-19 treatment.

**Table 4:** IFNs and ISGs related with ct value at admission. Multiple linear regression adjusted for age was performed. Log transformation was performed on IFNs or ISGs due to skewed distribution. IFNs or ISGs resulting in significant models are listed

Related IFNs and ISGs	Coefficients	p
*IFNL1*	-1.259	0.002
*IFNG*	-1.254	0.013
*MX1*	-2.137	0.004
*IFIT2*	-1.616	0.023
*IFIT3*	-1.502	0.002
*ISG15*	-1.466	< 0.001
*RSAD2*	-1.415	0.011
*IFI27*	-1.878	0.001
*IFI44L*	-1.377	0.011
*USP18*	-2.057	0.001

IFN: interferon, ISG: IFN-stimulated genes, *MX1*: Myxovirus resistance 1, *IFIT*: Interferon induced protein with tetratricopeptide repeats, *RSAD2*: Radical S-adenosyl methionine domain containing 2, *IFI27*: Interferon alpha induced protein 27, *IFI44L*: Interferon induced protein 44 like, *USP*: Ubiquitin specific peptidase

## Discussion

Our data revealed that upregulation of IFNs and ISGs occurred immediately after SARS-CoV-2 infection in the NP of mild-CoV2 + patients, in concert with the activation of IFN-related responses from M1 MФ. In addition, innate immune responses could be induced in the NP of severe-CoV2 + patients depending on viral RNA level during Omicron-dominant period and the prognosis of COVID-19 may depend on the induction of IFNs and ISGs in the NP of patients.

NP lymphoid tissue resembles nasal mucosa and has characteristics of lymph nodes and human respiratory viruses encounter host defense mechanisms in the NP. NP lymphoid tissue may therefore be an important organ in which an antiviral defense mechanism can be activated after SARS-CoV-2 infection, and host protection can be conferred by specialized innate immune responses of the NP capable of combating viral invasion [28]. Our data demonstrated that both IFNs and ISGs were induced in the NP of mild-CoV2 + patients and, in particular, transcription of *IFNA* and *IFNL* was elevated in the NP of severe-CoV2 + patients with induction of ISGs. While we still lack a full understanding about the role of local immune responses in the upper airway against SARS-CoV-2, previous studies have shown that SARS-CoV-2 suppresses activation of the innate immune system, in parallel with excessive activation of proinflammatory cytokines [6, 29, 30]. Innate immune responses are mediated by an increase of IFN production, which contributes to viral clearance in the respiratory tract [9, 31, 32]. Both type I and type III IFNs mediate the innate immune response in the respiratory epithelium and IFN deficiency has been linked to higher susceptibility to fatal lung infection [33, 34]. Moreover, the compensation of IFNs in upper airway can protect the host from SARS-CoV-2 and regulate subsequent activation of the defense mechanism in the lung [35]. We therefore hypothesized that this intimate induction of IFNs in the NP could potentially benefit a host’s respiratory tract and restrict SARS-CoV-2 replication through rapid upregulation of innate immune responses.

Our data revealed that the NP not only exhibited functional upregulation of genes related to “response to virus” and “defense response to virus,” but also demonstrated the activation of type I and type III IFNs and ISG signaling in CoV2 + patients. Both *IFNA* and *IFNL* may be dominant IFNs induced significantly in the NP of CoV2 + patients. In addition, *MX1*, *IFIT1*, *USP18*, and *RSAD2* were highly upregulated in the NP following SARS-CoV-2 infection. Induction of IFNs and ISGs may be involved in the suppression of viruses in the NP of severe-CoV2 + patients. Our understanding of the innate immune factors that contribute to the severity of COVID-19 in individuals infected with SARS-CoV-2 remains incomplete, but the development of severe lung inflammation does not appear to be attributable solely to viral load; other factors, such as a compromised IFNs, could be involved [36]. Our findings raise a key question regarding induction of innate immune responses in a host’s upper airway to enhance resistance to SARS-CoV-2 infection even in cases of severe COVID-19.

The present study provides insights into the relationships between innate immune factors and clinical parameters governing therapeutic outcome. We found that upregulated expression of *MX1*, and *RSAD2* was a prognostic factor of COVID-19 treatment. However, the induction of *MX1* and *RSAD2* was correlated with most clinical factors, including severity, age, hospitalization, initial chest X-ray abnormality, and SOFA score. In addition, CoV2 + patients with higher viral RNA level showed significant induction of IFNs and ISGs in their NP at initial stage of COVID-19. Collectively, we estimate that upregulation of innate immune factors in the NP may be beneficial for local viral control and protection against viral spread in individual patients. Multiple studies have recently described parallel impaired IFN activity and exacerbated inflammatory cytokine responses in severe-CoV2 + patients. However, our study revealed that IFN-related innate immune responses can be induced in the NP of severe-CoV2 + patients during the acute phase of infection, and upregulation of these innate immune factors can enhance the resistance to viral infection, resulting in improved prognoses.

We also assessed immune cell types with transcriptional alterations related to ISGs using the CIBERSORT program from bulk RNA sequencing data. Although this study did not involve single-cell RNA sequencing data, which provides unbiased transcriptional profiling of thousands of individual cells, this in silico flow cytometry method has the advantage of not requiring tissue disaggregation [25]. Additionally, it can examine 22 immune cell types simultaneously using a signature matrix, unlike conventional flow cytometry, which relies on small combinations of pre-selected marker genes [25].

Our sequencing data demonstrated that the transcriptional alteration related to M1 macrophages, DCs and CD4 + memory T cells were distinctive in the NP of CoV2 + patients. In addition, the induction of ISGs was significantly associated with higher transcriptions of M1 MФ and monocytes-related genes in the NP of CoV2+. It has been suggested that aberrant activation of immune cells and proinflammatory polarization of alveolar M1 macrophages are related to dysregulated local and mucosal inflammatory responses in the lungs of susceptible CoV2 + patients [37–39]. However, the current study implies that the increased transcriptional phenotypes related to M1 MФ can be combined with rapid viral clearance in the NP to promote the induction of ISGs during the early phase of SARS-CoV-2 infection.

The main limitation of our study is the small number of RNA sequencing samples used. Although we attempted to mitigate this limitation by conducting an experimental validation using real-time PCR with a large number of samples, the initial RNA sequencing data may not fully capture the complexity and heterogeneity of the studied population. Another limitation arises from the disparity in the sampling days between mild- and severe-CoV2 + patients. When COVID-19 progresses to pneumonia, it usually takes several days after the onset of symptoms. This might contribute to the difference in sampling time [40]. However, a specialized innate immune system at the NP combats invasion by SARS-CoV-2, and decreases the burden of disease in an infected host by increasing antiviral resistance [41, 42] and we have highlighted the importance of innate immune responses in the NP at early phase of COVID-19 during Omicron variant surge. There is increasing interest in IFN-related immune compromise mechanisms which is induced by respiratory virus including inborn errors or production of autoantibodies following COVID-19 infection [43, 44]. Unfortunately, we could not observe induction of type I IFNs transcriptions, and this adaptive autoimmunity may impair type I IFN-related antiviral immunity in a part of COVID-19 patients. However, we found that IFN-λ, IFN-γ and various ISGs were increased in the NP, the first target tissue of SARS-CoV-2 infection, and that Omicron-variant virus had a very subtle effect on weakening the innate immune mechanism. We estimate that this activation of the innate immune mechanism in the upper airway might provide good therapeutic results in the treatment of COVID-19.

Our results contribute to the current understanding of the intricate relationship between IFN-regulated innate immune responses in the NP and the therapeutic outcome of Omicron-variant SARS-CoV-2 infection even if it is severe disease. The human NP is a crucial anatomic structure for localized immune responses in upper airway depending on SARS-CoV-2 infection. Our data estimated that IFN-related innate immune responses can be induced in the NP during the early phase of COVID-19 regardless of severity of infection and activation of innate immune responses in upper airway may provide a better therapeutic outcome of COVID-19 treatment.

### Electronic supplementary material

Below is the link to the electronic supplementary material.

Supplementary Material 1

Supplementary Material 2

## Data Availability

The original contributions presented in the study are included in the article/supplementary material. The original bulk-seq data for are available at https://www.ncbi.nlm.nih.gov/geo/query/acc.cgi?acc=GSE239595.
