# Supplementary material for: Innate immune signatures in the nasopharynx after SARS-CoV-2 infection and links with the clinical outcome of COVID-19 in Omicron-dominant period
Source: Cell Mol Life Sci. 2024 Aug 22;81(1):364. doi: 10.1007/s00018-024-05401-1 (PMC11342914; doi:10.1007/s00018-024-05401-1)
Supplement: Supplementary file 1 — Supplementary Material 1 [file 18_2024_5401_MOESM1_ESM.docx]

**Supplementary figure1.** Heterogeneity of subjects for bulk RNA sequencing analysis (a) PCA plot illustrates the variability in gene expression profiles among subjects. (b) The heatmap displays the expression levels of the top 35 upregulated and 35 downregulated genes in CoV+ patients compared to controls. Gene expression levels are presented as z-scores and log fold changes. Each row corresponds to a gene, and each column represents a subject. The color gradient indicates the magnitude of gene expression changes, providing a visual representation of the heterogeneity among subjects in response to COVID-19 infection.


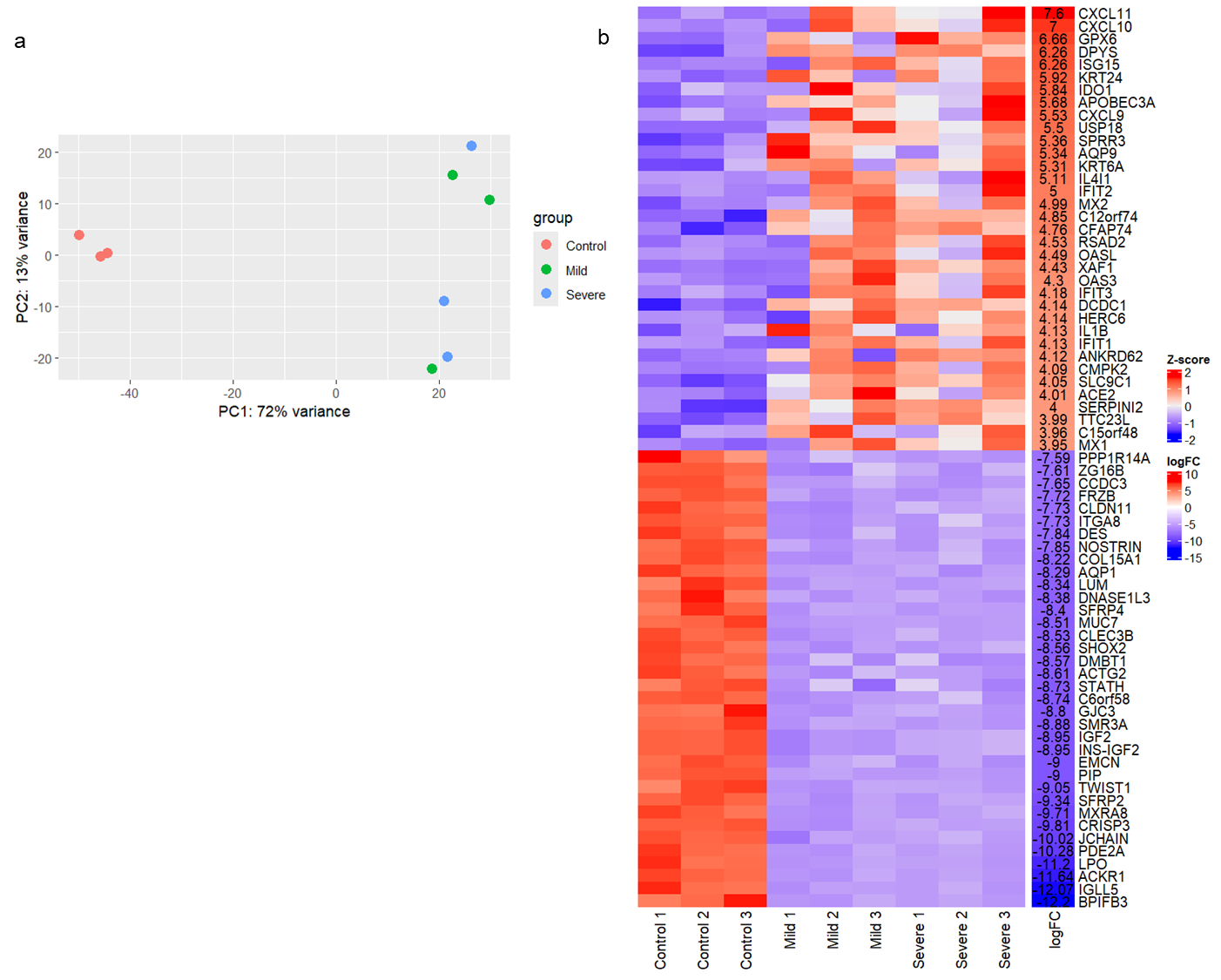


**Supplementary figure 2.** Enrichment analysis of downregulated genes based on gene ontology(GO)–biological process analyses. The figure presents the top 10 GO categories from an enrichment analysis of downregulated genes in CoV+ patients compared to healthy controls (n=3 for each group).

**
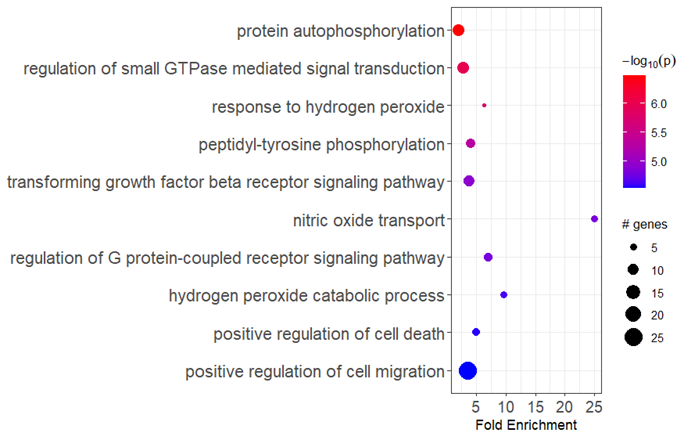
**
