# Supplementary material for: Innate immune signatures in the nasopharynx after SARS-CoV-2 infection and links with the clinical outcome of COVID-19 in Omicron-dominant period
Source: Cell Mol Life Sci. 2024 Aug 22;81(1):364. doi: 10.1007/s00018-024-05401-1 (PMC11342914; doi:10.1007/s00018-024-05401-1)
Supplement: Supplementary file 2 — Supplementary Material 2 [file 18_2024_5401_MOESM2_ESM.doc]

**Supplementary Table 1. The list of tagman primers**

| **Gene** | **Amplicon** | **Assay ID** | **Dye** |
| --- | --- | --- | --- |
| ***IFNA*** | 146 | Hs03044218_g1 | FAM-MGB |
| ***IFNB*** | 73 | Hs01077958_s1 | FAM-MGB |
| ***IFNG*** | 73 | Hs00989291_m1 | FAM-MGB |
| ***IFNL1*** | 101 | Hs00601677_g1 | FAM-MGB |
| ***IFNL2/3*** | 167 | Hs00820125_g1 | FAM-MGB |
| ***IFNL4*** | 77 | Hs04400217_g1 | FAM-MGB |
| ***CXCL10*** | 98 | Hs00171042_m1 | FAM-MGB |
| ***TNF*** | 80 | Hs00174128_m1 | FAM-MGB |
| ***IL1B*** | 91 | Hs01555410_m1 | FAM-MGB |
| ***MX1*** | 53 | Hs00895608_m1 | FAM-MGB |
| ***IFIT1*** | 134 | Hs03027069_s1 | FAM-MGB |
| ***IFIT2*** | 115 | Hs01584837_s1 | FAM-MGB |
| ***IFIT3*** | 137 | Hs01922752_s1 | FAM-MGB |
| ***RSAD2*** | 76 | Hs00369813_m1 | FAM-MGB |
| ***USP18*** | 98 | Hs07289021_m1 | FAM-MGB |
| ***ISG15*** | 100 | Hs00192713_m1 | FAM-MGB |
| ***IFI27*** | 68 | Hs01086373_g1 | FAM-MGB |
| ***IFI44L*** | 78 | Hs00915292_m1 | FAM-MGB |

**Supplementary Table 2. Correlations of IFNs and cytokines with clinical factors**

| Factors | *IFNA* | *IFNB* | *IFNL1* | *IFNL2/3* | *IFNL4* | *IFNG* | *CXCL10* | *TNFA* | *IL1B* |
| --- | --- | --- | --- | --- | --- | --- | --- | --- | --- |
| Severity | 0.142 | 0.035 | -0.317* | -0.326* | -0.186 | -0.272* | -0.315* | 0.023 | -0.188 |
| Sex | -0.081 | 0.162 | -0.019 | -0.060 | -0.187 | 0.023 | -0.088 | 0.091 | -0.032 |
| Age | -0.044 | -0.041 | -0.317* | -0.331** | -0.394** | -0.325* | -0.484*** | 0.003 | -0.260* |
| Number of vaccine doses | -0.043 | -0.371** | 0.199 | 0.036 | 0.186 | 0.051 | 0.092 | -0.049 | 0.078 |
| Previous history of COVID-19 | 0.156 | -0.050 | -0.116 | -0.061 | -0.073 | -0.064 | 0.056 | 0.023 | 0.183 |
| Hospitalization | 0.078 | 0.093 | -0.435*** | -0.266* | -0.400** | -0.262* | -0.688*** | 0.119 | -0.373** |
| Length of in-hospital stay | 0.401** | 0.004 | -0.155 | -0.064 | -0.088 | -0.086 | 0.059 | 0.276* | -0.095 |
| Initial chest X-ray abnormality | 0.062 | 0.037 | -0.427** | -0.323* | -0.104 | -0.357** | -0.175 | -0.225 | -0.211 |
| Duration of supplemental oxygen therapy | 0.399** | 0.045 | -0.143 | -0.088 | -0.082 | -0.106 | 0.086 | 0.229 | -0.095 |
| SOFA score | 0.249 | 0.042 | -0.350** | -0.252 | -0.029 | -0.271* | -0.228 | -0.089 | -0.162 |
| Ct value at admission | -0.170 | -0.145 | -0.156 | -0.111 | -0.094 | -0.075 | -0.195 | -0.187 | -0.052 |

Data are pearson correlation coefficient. Asterisks represent significant differences (P-value < 0.05, **P-value < 0.01, ***P-value < 0.001). *IFNA, IFNB, IFNL1, IFNL2/3, IFNL4, IFNG, CXCL10, TNFA*, and *IL1B* are log transformed due to skewed distribution. IFN: interferon, CXCL: chemokine (C-X-C motif) ligand, TNF: tumor necrosis factor, IL: interleukin.

**Supplementary Table 3. Correlations of ISGs with clinical factors**

| Factors | *MX1* | *IFIT1* | *IFIT2* | *IFIT3* | *RSAD2* | *USP18* | *ISG15* | *IFI27* | *IFI44L* |
| --- | --- | --- | --- | --- | --- | --- | --- | --- | --- |
| Severity | -0.286* | -0.141 | -0.053 | -0.120 | -0.292* | 0.010 | -0.272* | -0.265* | -0.282* |
| Sex | -0.018 | 0.078 | 0.031 | 0.038 | -0.057 | 0.018 | -0.014 | 0.000 | -0.094 |
| Age | -0.346** | -0.148 | -0.258* | -0.234 | -0.310* | -0.181 | -0.383** | -0.301* | -0.336** |
| Number of vaccine doses | 0.132 | 0.110 | 0.095 | 0.079 | 0.165 | 0.104 | 0.095 | 0.141 | 0.103 |
| Previous history of COVID-19 | -0.108 | -0.118 | 0.066 | -0.047 | -0.124 | -0.014 | -0.060 | -0.151 | -0.118 |
| Hospitalization | -0.485*** | -0.089 | -0.272* | -0.214 | -0.433*** | -0.149 | -0.462*** | -0.334** | -0.518*** |
| Length of in-hospital stay | -0.084 | -0.042 | 0.236 | 0.048 | -0.148 | 0.112 | 0.048 | -0.131 | -0.164 |
| Initial chest X-ray abnormality | -0.394** | -0.358** | -0.132 | -0.188 | -0.358** | -0.090 | -0.306* | -0.384** | -0.164 |
| Duration of supplemental oxygen therapy | -0.087 | -0.075 | 0.225 | 0.017 | -0.121 | 0.087 | 0.038 | -0.149 | -0.114 |
| SOFA score | -0.346** | -0.220 | -0.064 | -0.106 | -0.361** | 0.039 | -0.252 | -0.315* | -0.272* |
| Ct value at admission | -0.307* | -0.166 | -0.225 | -0.255 | -0.274 | -0.315* | -0.332* | -0.233 | -0.081 |

Data are pearson correlation coefficient. Asterisks represent significant differences (P-value < 0.05, **P-value < 0.01, ***P-value < 0.001). *MX1, IFIT1, IFIT2, IFIT3, RSAD2, USP18, ISG15, IFI27,* and *IFI44L* are log transformed due to skewed distribution. IFI: Interferon Induced Protein with Tetratricopeptide Repeats, RSAD: Radical S-adenosyl methionine domain containing, USP: ubiquitin specific peptidase, ISG: Interferon-stimulated gene, IFI: Interferon Alpha Inducible Protein
